# North-African doctors as second victims of medical errors: a cross sectional survey

**DOI:** 10.1186/s12888-022-04049-0

**Published:** 2022-06-20

**Authors:** Imen Ben Saida, Sabil Grira, Radhouane Toumi, Amani Ghodhbani, Emna Ennouri, Khaoula Meddeb, Helmi Ben Saad, Mohamed Boussarsar

**Affiliations:** 1 University of Sousse, Faculty of Medicine of Sousse, 4000 Sousse, Tunisia; 2 Farhat Hached University Hospital, Medical Intensive Care Unit, Research Laboratory “Heart Failure”, LR12SP09, 4000 Sousse, Tunisia; 3 Farhat Hached University Hospital, Laboratory of Physiology and Functional Explorations, Research Laboratory “Heart Failure”, LR12SP09, 4000 Sousse, Tunisia

**Keywords:** Medical error, Second victim, Coping strategy, Changes in practice, North-African physicians

## Abstract

**Introduction:**

Physicians involved in medical errors (MEs) can experience loss of self-esteem and negative psychological experiences. They are called “second victims” of the ME.

**Aims:**

To *i)* describe the profile, the types and the severity of MEs, and *ii)* explore the psychological impact on “second victims” to better understand how they cope.

**Methods:**

It was a cross sectional retrospective study conducted from March to August 2018. All physicians working at Farhat Hached and Sahloul University hospitals were asked to complete a questionnaire about their possible MEs. The impact of MEs was evaluated using the Impact of Event Scale-Revised (IES-R) (scoring, 0–88) (subscales ranges; intrusion, (0–32); avoidance, (0–32); hyperarousal, (0–24)). The diagnosis of post-traumatic stress disorder (PTSD) was made when the total IES-R score exceeded 33. The coping strategies were evaluated using Ways of Coping Checklist Revised (WCC-R) scale (scoring, problem-focused, (10–40); emotion focused, (9–36); seeking social support, (8–32)).

**Results:**

Among 393 responders, 268(68.2%) reported MEs. Wrong diagnosis (40.5%), faulty treatment (34.6%), preventive errors (13.5%) and faulty communication (6.4%) were the main frequent types of MEs. The most common related causes of MEs were inexperience (47.3%) and job overload (40.2%). The physicians’ median (range) score of the IES-R was 19(0–69). According to the IES-R score, the most frequent psychological impacts were median (range): intrusion, 7(0–28) and avoidance symptoms, 7(0–24). PTSD symptoms affected 23.5% of physicians. Female sex and serious MEs were identified as predictors of PTSD. On the WCC-R check list, coping was balanced between the three coping strategies median (range), problem focused, 28.5(10–40); emotion-focused, 24(9–36) and seeking social support 21(8–32).

**Conclusion:**

There is a relatively high impact of ME within these North-African university hospital physicians. Coping was balanced within different three strategies as reported worldwide. Physicians adopted more likely constructive changes than defensive ones.

**Supplementary Information:**

The online version contains supplementary material available at 10.1186/s12888-022-04049-0.

## Introduction

Despite rigorous attempts to improve the safety of health care, medical errors (MEs) are still common with high additional health care cost, morbidity and mortality [1, 2]. In 2000, The Institute of Medicine in its report “To Err is Human: Building a Safer Health System” estimated that the death rate due to MEs in United States was 44,000 to 98,000 [1]. Twenty years later, the World Health Organization (WHO) estimated that in high-income countries one in every 10 patients is harmed while receiving hospital care and each year 134 million adverse events occur in hospitals in low- and middle-income countries, due to unsafe care, resulting in 2.6 million deaths [3]. Another report estimated that these adverse events result in 23 million disability-adjusted life years lost per year in the world [4]. MEs is a universal issue however it remains underreported in low- and middle-income countries. Epidemiological data regarding the incidence, type, causes and consequences of MEs from North-Africa are scarce. As a part of a project built by the World Alliance for Patient Safety in collaboration with the World Health Organization in 2007, a prospective study in Moroccan medical intensive care units found an overall ME incidence at 7.7 per 1000 patient-days [5].

Even highly trained physicians can experience MEs potentially leaving them traumatized for days, weeks, and even years after an event [6]. The physician traumatized by a ME is considered the “second victim”, noting that the first victim is the patient, the third victim is the hospital reputation, and the fourth victim is the patient harmed subsequently [7].

Since the original description by Albert Wu [8], some physicians dislike the idea of being a victim, as the word denotes a degree of passivity and helplessness [9]. MEs related to patient tragedies such as loss of life, harm from violence, the first experience of losing a patient, or a traumatic painful experience can affect even the most resilient health care professional [10]. Many “second victims” of MEs may suffer career-related stress and anxiety. They are more likely to report symptoms of burnout and depression, something that will in turn lead to higher risks of making new MEs, in a reciprocal circle [11, 12]. Furthermore, empathy and a tendency to minimize the event in question might affect decision-making in the future [13, 14]. For that, some physicians may refuse to accept similar patients because of that fear, which can be considered as “negative defensive medicine” [15]. In fact, the second victim experience depends on many variables such as the type of the error, severity of injury to the patient, emotional response of the healthcare practitioner and support or blame by colleagues or mentors. Developed countries implemented institutional programs to help mitigate the negative impact of the second victim phenomenon [7]. Since this topic is still taboo in North-African countries and very little attention is dedicated to healthcare professionals involved in MEs, the extent of second victim experience is unknown.

In order to better understand how North-African “second victims” cope and which support can be provided for them; it is important to ***i)*** identify their profile; ***ii)*** describe the types and the severity of MEs they committed; and ***iii)*** explore the psychological impact of these MEs.

## Participants and methods

### Study design

It was a cross-sectional retrospective study. The study was conducted from March to August 2018 with all senior and junior physicians of different departments in two tertiary university hospitals (Farhat Hached and Sahloul Hospitals, Sousse, Tunisia). The research and Ethics committee of the University Hospital Farhat Hached, Sousse approved the study and waived the need for a written informed consent as the study was a cross-sectional retrospective one including physicians who were all informed about the purpose of the survey. The voluntary nature of their participation and the confidentiality of each participants’ answers were guaranteed by asking participants to fill out the questionnaire in private, place them in a dedicated box left in each department and collected a week later by the co-investigators (SG, AG).

### Population

The present study is part of a project involving two parts. The first is the objective of this study. The second part will aim to identify factors that predict Tunisian second victims’ coping strategies.

The population of the study consisted of all senior and junior physicians working in the aforementioned two tertiary university hospitals, which are Tunisian tertiary-level major academic university hospitals with a total of 1330 beds.

### Sample size

To obtain representative and reliable data, the required sample size was estimated using the following equation [16]: n = (Z_α/2_^2^ p (1-p))/∆^2^. “***Z***_***α/2***_” (=2.33) was the normal deviate for a one-tailed hypothesis at a 1% level of significance; “***p***” (=0.39) was the frequency of “serious” MEs reported in a previous study involving 439 pediatric attending physicians and 118 residents [17], “***∆***” was the precision (arbitrarily fixed at 6.0%). Using the aforementioned equation, the estimated sample size was 360 physicians. The assumption of 20% of refuse gives a revised sample of 450 physicians (450 = 360/(1–0.20)).

### Collected data

Data were collected via a questionnaire, developed after reviewing the relevant literature on psychological impact of MEs and coping strategies, using internationally validated scales. The self-report questionnaire includes the following five parts.

1. *Demographic data:* the following data were assessed: age (years), sex (male/female), marital status (single, married, divorced, widower), type of practitioner [junior physicians (interns and residents); senior physicians (experienced physicians having completed their training)]; department (medical, surgical, intensive care unit), and years of experience.
2. *MEs:* respondents were asked to answer “yes” or “no” to the question on whether or not they knew about the term of “second victim”. They were also asked to indicate whether they had ever been personally involved in MEs in their career. For those answering positively to any ME, the survey continued, and the respondents were asked to think about the event that they had perceived as the worst. MEs were assessed based on the studies conducted by Leape et al. [14] and Wu et al. [15], which identified four major categories of MEs, largely described in Table 1 Based on the study of Wu et al. [15], causes of MEs were classified into the four following categories: inexperience, faulty communication, job overload, and complex case. Severity of reported MEs was assessed according to the degree of harm into the following five categories [19]: “no harm”, “mild harm”, “moderate harm”, “severe harm”, and “not assessable”. For practical and statistical reasons, “no harm” and “mild harm” were combined into “minor” harm, while “moderate harm” and “severe harm” were considered as “serious” harm that may have caused or resulted in the patient’s death. Not assessable MEs were excluded from statistical analysis.
3. *Impacts of MEs:* Several instruments have been developed to measure PTSD symptoms after traumatic events, but the IES-R was the first instrument developed for this purpose and the most widely used self-report scale.

**Table 1:** Categories of medical errors and severity of reported errors

Category		Description
**Medical errors** [14, 18]		
***Diagnostic***		.Incorrect choice of therapy .Wrong diagnostic test .Misdiagnosis leading to an incorrect choice of therapy .Use of outmoded tests or therapy .Misinterpretation of test results .Failure to do the indicated diagnostic tests .Failure to act on results of monitoring or testing
**Treatment**		.Reception of the wrong drug .Inappropriate care .Error in the administration of the treatment .Error in the dose or method of using a drug leading to the patient’s death .Delay in treatment
**Preventive**		.Failure to provide prophylactic treatment .Inadequate monitoring or follow-up of treatment
**Other**		.Failure in communication (inadequate informed consent) .Equipment or system failure
**Severity of reported medical errors (harms)** [19]		
**No harm**	**Minor errors**	.Any medical error that had the potential to cause harm but was prevented, resulting in no harm to patient
**Mild harm**		.Any unexpected or unintended incident that required extra observation or minor treatment and caused minimal harm to one or more persons
**Moderate harm**	**Serious errors**	.Any unexpected or unintended incident that resulted in further treatment, possible surgical intervention, cancelling of treatment, or transfer to another area, and which caused short-term harm to one or more persons
**Severe harm**		.Any unexpected or unintended incident that caused permanent or long-term harm to one or more persons
**Not assessable**	**Excluded**	

IES-Revaluates the subjective response to a specific traumatic event in the adult population, especially in the response sets of the following three items: i) intrusion subscale [intrusive thoughts, nightmares, intrusive feelings and imagery, dissociative-like re-experiencing (items 1, 2, 3, 6, 9, 14, 16, 20)], ii) avoidance subscale [numbing of responsiveness, avoidance of feelings, situations, and ideas (items 5, 7, 8, 11, 12, 13, 17, 22)], and iii) hyperarousal subscale [anger, irritability, hyper-vigilance, difficulty concentrating, heightened startle (items 4, 10, 15, 18, 19, 21)].

Scoring of the IES-R includes a total score (ranging from 0 to 88) and three reflecting intrusion, (0–32); avoidance, (0–32) and hyperarousal, (0–24)).

The French version used in the present study was validated in 2003 [20] with good internal validity (alpha coefficients ranging from 0.81 to 0.93) and test-retest reliability (correlation coefficients ranging from 0.71 to 0.76) to assess post-traumatic stress reactions. The three-factor solution was validated with a total explained 56% of the variance.

The strengths of this tool are that it is short, simply administered and scored. It corresponds better with the “diagnostic and statistical manual of mental disorders” criteria for PTSD and can easily be used repetitively.

The diagnosis of post-traumatic stress disorder (PTSD) was made when the total IES-R score exceeded 33 [21].

4) *Disclosure of MEs:* this part identifies whether physicians ever disclosed a ME or not, and how satisfied they were with past disclosure experience.
5) Coping with MEs: coping was evaluated using a French version of the “Ways of Coping Checklist Revised (WCC-R)” scale validated by Cousson et al. with good psychometric properties [22]. It is a brief (5 to 10 minutes) self-reported tool [23]. WCC-R includes 27 items. Respondents use a 4-point Likert-type scale ranging from No, 1 to Yes, 4 except item 15, which is rated in reverse (No, 4; Yes, 1). The component analysis identified three principal coping strategies [22], the scores are obtained by summing the scores of items corresponding to each strategy: i) Coping focused on the problem [to cope with the problem that causes distress, to be followed in action, to fight, to feel strong, and to find a solution and to learn from the mistakes (items 1, 4, 7, 10, 13, 16, 19, 22, 25, 27)], ii) Coping focused on emotions [managing the emotional distress caused by error, accepting responsibility for the mistake and recommending practice changes to reduce future errors, feelings of weakness, guilt, self-criticism, hope for miracles, change and need to forget (items 2, 5, 8, 11, 14, 17, 20, 23, 26)], and iii) Seeking social support [it’s not only about notions of informal and material support, but also about emotional support (items 3, 6, 9, 12, 15, 18, 21, 24)]. Several coping scores were calculated. The WCC-R subscales are ranging as follows (problem-focused, (10–40); emotion focused, (9–36); seeking social support, (8–32)). A score closer to 40 means a higher probability of using the corresponding coping strategy while a score closer to 10 means a lesser likelihood of using it.

IES-R and WCC-R respective reliability and validity in the present study are assessed and displayed as supplementary material.

### Statistical analysis

The Kolmogrov-Smirnov test was used to analyze the distribution of variables. Results were expressed as mean ± standard deviation (SD) when the distribution was normal, and variances were equal. If not, results were expressed by their medians (range). Categorical data were expressed by their relative proportions. For categorical data, chi-square tests were used. For continuous data, Student’s t or Mann-Whitney U tests were used if the data were respectively normally or non-normally distributed. Data coding and data entry were performed and files with missing data were excluded from further analysis. Statistical analyses were performed using the statistical software package SPSS 20.0. Significance threshold was set at 0.05.

## Results

**Fig. 1:**
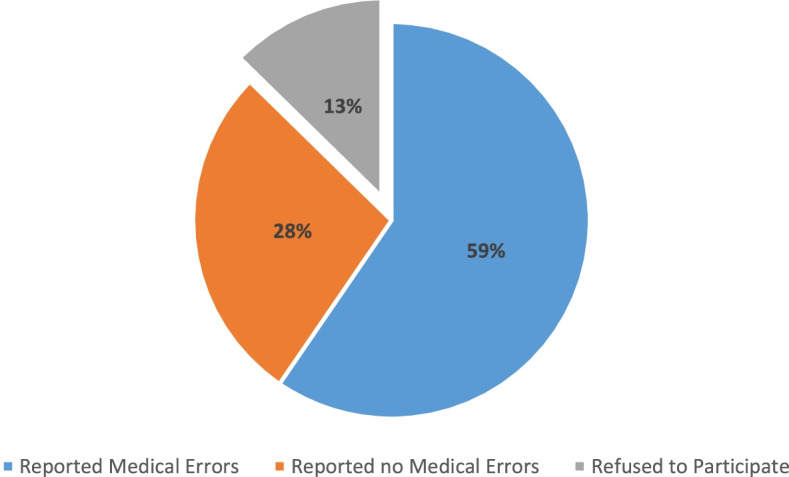
Respondents’ distribution regarding refuse, reporting or non-reporting medical errors

Among the initial sample of 600 physicians, 150 were ineligible because they had retired or were not clinically active, resulting in 450 eligible physicians. Among the latter group, surveys were completed by 393 (response rate of 87.5%). Two hundred sixty-eight (68.2%) respondents reported a ME in their practice (Fig. 1**)**.

The baseline characteristics of respondents were summarized in Table 2.

**Table 2:** Respondent’s characteristics divided by groups: group 1 (physicians who reported medical errors, *n* = 268); group 2 (physicians who reported no medical errors, *n* = 125)

Items	Total sample (*n* = 393)	Group 1 (*n* = 268)	Group 2 (*n* = 125)	***P***-value
**Sex**				
Male	148 (37.7)	102 (38.1)	46 (36.8)	0.810
Female	245 (62.3)	166 (61.9)	79 (63.2)	
**Age** (Years)	30 ± 7	31 ± 7	29 ± 7	0.025
**Years in practice** (Years)	5.4 ± 7.1	5.0 ± 7.1	4.3 ± 6.9	0.037
**Marital status**				
Single or divorced	260 (66.2)	164 (61.2)	96 (76.8)	0.020
Married	133 (33.8)	104 (38.8)	29 (23.2)	
**Type of practitioner**				
Senior doctor	88 (22.4)	72 (26.9)	16 (12.8)	0.020
Junior doctor	305 (77.6)	196 (73.1)	109 (87.2)	
**Type of department**				
Medical	267 (67.9)	183 (31.7)	84 (67.2)	0.830
Surgical/intensive care	126 (32.1)	85 (68.3)	41 (32.8)	

Quantitative and categorical data were mean ± SD and number (%), respectively. Comparison between the 2 groups (Chi-square test or Student’s t-Test)

**Fig. 2:**
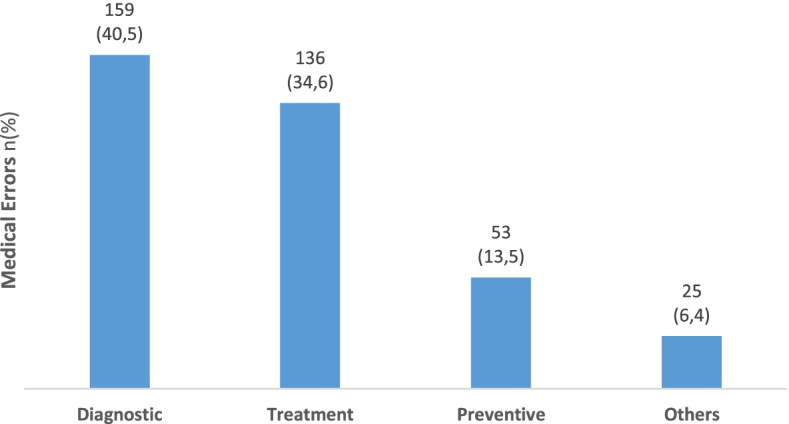
Types of reported medical errors

The number of reported MEs and the percentage accounted for each type of ME are listed in Fig. 2. The most frequently reported type of MEs was a wrong diagnosis (40.5%).

Physicians mostly attributed MEs to more than one cause. The following causes were reported: inexperience (47.3%), job overload (40.2%), case complexity (27%), and faulty communication (14%). Forty-one (15.3%) physicians reported MEs resulted in “severe harm”.

Out of 253 respondents, 45.1 and 54.9% reported serious and minor MEs, (Table 3). There were no statistically significant differences in sex between serious and minor MEs, however, female physicians reported more serious MEs. There was a statistically significant difference in the level of harm reported by junior and senior physicians (*p* = 0.011).

**Table 3:** Association of demographic factors with levels of harm (*n* = 253)

	Minor harm (***n*** = 139)	Serious harm (***n*** = 114)	***P-***value
**Sex**			
Male	54 (38.8)	42 (36.8)	0.743
Female	85 (61.2)	72 (63.2)	
**Type of practitioner**			
Junior doctors	109 (78.4)	73 (64)	0.011
Senior doctors	30 (21.6)	41 (36)	
**Department**			
Medical	100 (71.9)	74 (64.9)	0.230
Surgical/intensive care	39(28.1)	40 (35.1)	

Data were number (%). Chi-square test: comparison between the 2 groups

Regardless of the level of training, nearly all respondents reported that they had never heard the term “second victim” before. Only, 62 (16%) participants reported knowing about it. Two hundred and thirty-five participants (87.7%) reported disclosing their MEs. Erring physicians found encouragement and support in talking to their peers in 68% of cases (*n* = 182).

Physicians were found to have discussed MEs informally with peers more than those who had reported MEs to their chair or patient. Only twenty-seven physicians (10.1%) revealed the ME to the patient and asked for forgiveness. Sixty-six percent (*n* = 177) of physicians who encountered an error were satisfied about disclosing a ME, others reported that disclosure led to shame (*n* = 70), to legal action (*n* = 10), and made them worry about being blamed (n = 17). The overall median IES-R score for all participants was 19, with a range of 0–69. The most frequent symptoms of subjective trauma distress sub-scale were median (range): intrusion, 7(0–28) and avoidance, 7(0–24) and hyperarousal, 4(0–21).

Table 4 exposes factors influencing the impact of reported MEs on physicians. It shows that female respondents reported a lot more distress than males, with reference to IES-R total score. Physicians experience the biggest impact after an incident with serious harm with a statistically significant relationship between levels of harm and IES-R total.

**Table 4:** IES-R total score according to the respondents’ characteristics (*n* = 253)

Characteristics	IES-R total score	***P***-value
**Age**		
≤ 30 years	17 (0–69)	0.258
> 30 years	21 (0–69)	
**Sex**		
Male	15 (0–59)	0.015
Female	20.5 (1–69)	
**Marital status**		
Single or Divorced	16 (0–69)	0.196
Married	21 (0–65)	
**Type of practitioner**		
Junior doctor	16.5 (0–69)	0.131
Senior doctor	21 (0–65)	
**Level of harm**		
Minor	13 (0–65)	0.000
Serious	22 (0–69)	

IES-R (impact of event scale-revised) was median (range). Mann-Whitney U test: comparison of IES-R total score between groups for each characteristic

Only 63(23.5%) physicians reported symptoms of ‘probable’ PTSD. Females and physicians experiencing serious MEs were more likely to develop PTSD syndrome after ME (Table 5).

**Table 5:** Factors associated with post-traumatic stress disorder (PTSD) among physicians involved in medical errors (*n* = 268)

	No PTSD (***n*** = 205)	Probable PTSD (***n*** = 63)	***P-value***
**Sex**			
Female	118 (57.6)	48 (76.2)	0.008
**Marital status**			
Single or divorced	128 (62.4)	36 (57.1)	0.451
Married	77 (37.6)	27 (42.9)	
**Type of practitioner**			
Junior physicians	151 (73.6)	45 (71.4)	0.727
Senior physicians	54 (26.3)	18 (28.6)	
**Department**			
Medical	140 (68.3)	43 (68.2)	0.123
Surgical/intensive care	65 (31.7)	20 (31.8)	
**Years in practice** (Years)	5.75 ± 7.04	6.48 ± 7.26	0.479
**Level of harm**			
Minor	118 (60.8)	21 (35.6)	0.001
Serious	76 (39.2)	38 (64.4)	

Quantitative and categorical data were mean ± SD and number (%), respectively. No PTSD: impact of event scale-revised (IES-R) ≤ 33. Probable PTSD: IES-R > 33.Comparison between the 2 groups (Chi-square test or Student’s T-Test)

Using the WCC-R scale, coping was balanced between the three coping strategies median (range), problem focused, 28.5(10–40); emotion-focused, 24(9–36) and seeking social support, 21(8–32).

IES-R and WCC-R in the present study had good reliability with Cronbach’s alpha or coefficient alpha respectively at 0.93 and 0.843. Content validity (redundancy) and convergent validity were satisfactory for both scales (see supplementary material).

MEs led to significant change in respondents’ learning behaviors. Almost all physicians reported some change in their practice after being involved in a ME. The most frequently reported changes were reading (79.5%), asking superiors (71.6%), and paying more attention to details (70%). Only 5% reported one or more defensive changes. A summary of constructive and defensive changes reported by physicians was reported in Table 6.

**Table 6:** Changes in practice described by physicians “second victims” (*n* = 268)

Constructive changes (95% of the total sample)	
**Increased information seeking**	
Ask superiors	192 (71.6)
Ask peers	174 (64.9)
Read	213 (79.5)
**Increased vigilance**	
Pay more attention to details	190 (70)
Trust others’ judgment less	43 (16)
Personally confirm data	143 (53.4)
**Defensive changes (5%)**	
Keep mistakes to oneself	32 (11.9)
Avoid similar patients	18 (6.7)

Data were number (%)

## Discussion

The main findings of the present study, which investigates the impacts of MEs on a sample of North-African physicians, were the following: ***i)*** 68.2% of physicians encountered a ME, ***ii)*** 84% of physicians reported not knowing the term “second victim”, ***iii)*** wrong diagnosis, faulty treatment, preventive errors and faulty communication were the main types of MEs, ***iv)*** the two common causes of MEs were inexperience and job overload, ***v)*** female sex and involvement in serious MEs were identified as predictors of PTSD, and ***vi)*** MEs had significant impacts on physicians’ behavior.

In 2000, Albert Wu [8] coined the term “second victim” to describe the emotional response of clinicians to MEs. This term has since been used to describe healthcare providers who experience difficulties to cope with their emotions after MEs and who may suffer in silence [10, 24]. According to Denham et al. [25], care providers can be psychologically harmed by unintentional MEs while trying to help patients. If harmed patients and their families are considered “first victims”, “second victims” are the caregivers involved in those unintentional MEs. Furthermore, he considered as “third victims” the health care organization harmed by leaders’ behavior.

In the present study, 57 of respondents refused to answer the questionnaire. It may be that professionals who responded had been notably more affected by serious/severe events and their related problems than their non-responding colleagues. However, non-responders could also be considered as the most severely affected group by traumatic events; they may have found the survey too personal or emotionally disturbing hence their abstinence from participating.

### Frequency of MEs

MEs are common, and most clinicians are likely to make them at least once in their careers [26]. In the present study, only 68.2% of respondents reported prior involvement in a ME. This could be integrated as a severe psychological impact in those responders reporting no errors, meaning they never coped with their errors. As those responders were younger and less experienced this result could be otherwise related to the short period of exposure. First, this was in line with a previous study reporting a frequency of 67% [27]. Second, lower frequencies of MEs were reported by some authors [10, 28]. For example, Lander et al. [28] analyzed otolaryngologists’ responses to ME and reported 10.4% of MEs, and Scott et al. [10] who analyzed psychological, emotional and professional support for health care providers noted 30%. Third, a higher frequency was reported by Garbutt et al. [17] who noted that 97% of pediatricians were involved in serious MEs. Discrepancies in rates could be explained by sincerity in response to questionnaires, methodological differences, and cultural influence in reporting errors. In this study, 42.5% of respondents reported prior involvement in serious MEs. Similar findings were noted in different studies reporting serious MEs resulting in deaths in 31% [18], 34% [29] and 39% [30] of cases.

### Causes of MEs

In this study, inexperience (47.3%) and job overload (40.2%) were the most selected reasons for MEs reported by physicians. This finding is consistent with results from a survey conducted by Wu et al. [18] who highlighted that 54% of house officers attributed MEs to inexperience and 51% reported job overload. Other studies provided same assessment of causes in the analysis of MEs [31, 32]. These studies stated that lack of experience was the most prevalent which was reported by 52% [31] and 39.2% [32] of participants.

Poor communication is an important cause of MEs in health care systems [15]. In this study, 14% of junior and senior physicians attributed their MEs to faulty communication. In fact, routine team checklist briefings could have a positive effect on team communication and teamwork and therefore reducing ME.

### Disclosure of MEs

Instead of concealing MEs, honest and transparent disclosure is emerging as the most appropriate way to deal with them [33]. Disclosure concerned 87.7% of respondents in this study. More than half of respondents talked to peers after a severe ME. This datum confirms previous findings [33, 34] suggesting that most physicians think they should share the story with a trusted colleague. Disclosing ME to patients is a challenging communication task. However, most physicians have never been trained in what to say, and how to say it [35]. In the present study, disclosing ME to patients was reported by 10.1% of physicians. This finding is consistent with reports suggesting that physicians are reluctant to tell patients about MEs because disclosure to patients requires a specific set of communication skills frequently lacking in physicians’ training [36]. This result is in line with that of some related studies [15, 37]. Legal and ethical experts, however, suggest that patients should generally be told about MEs [38]. Hilfiker [39] argues that disclosing a ME to the patient may be the only way for the physician to achieve a sense of absolution. Majority of respondents who disclosed MEs were generally satisfied which is in line with a finding of a study concluding that many physicians sought solace by discussing an error [40].

Multiple barriers may inhibit physicians from disclosure such as blame, legal action, loss of self-confidence, and reputation damage [41]. Thirty-four percent of responders were dissatisfied after disclosing MEs in the present study and the main reasons were shame (72.2%), legal action (10.3%), and worry about blame (17.5%). Those results can be explained by the culture of blame and punishment dominating in developing countries and the lack of supportive organizational programs. The same results were reported by Wu et al. [42] who noted that disclosing MEs exposed physicians to the risk of malpractice suits and public reputation damage.

### Impact of MEs

Physicians may suffer from severe distress, anxiety, guilt, shame, self-doubt, loss of self-esteem which may harm the quality of their professional and private life [43, 44]. These emotions can lead to a permanent emotional scar and a disruption in the therapeutic relationship with patients [45]. As stated below, the high proportion of responders reporting no errors may refer to an underlying severe psychological impact, meaning they never coped with their errors. The overall median IES-R score for our respondents was 19. However, measuring the IES-R score retrospectively may underestimate it. In fact, Van Gerven et al. [46] noticed a decrease from 17.72 at time of the incident to 8.99 at the time of the questionnaire. The present study, along with other previous reports in literature, confirmed that individual characteristics influence the impact and that females tend to report significantly more distress than males. Those results are in line with previous reports [11, 18, 46–49]. Seys et al. [47] explained the aforementioned finding by the fact that female “second victims” are more concerned about losing their confidence and being blamed, and experience more loss of reputation from their colleagues.

The degree of harm also influenced the impact of ME in the present study. These findings are in line with those of Van Gerven et al. [46] who mentioned that physicians experience the most severe impact after a serious harm incident. There is however a disagreement in the literature as to whether the impact on “second victims” depends on the severity of the event [48, 50] or remains the same no matter what happened [11].

The “second victim” can live constant emotional distress and can develop PTSD [44, 51, 52]. PTSD is a psychological disorder that could result from stressful events happening during the daily practice of physicians. Its symptoms may include insomnia, nightmares, reliving the incident repeatedly, loss of trust by their colleagues, lack of self-confidence, and fear of making another error [44, 53]. The current study examined the consequences of MEs on physicians. The data in the literature are extremely divergent concerning the prevalence of psychiatric disorders occurring after a traumatic event, which depends on the measuring instruments used as well as the events experienced [54–56]. On the one hand, our frequency of PTSD (23.5%) was comparable with reports from previous hospital studies **(*eg***;17% of Germanium psychiatric hospital staff [55], 18.4% [54]). On the other hand, no cases were found in a Sweden study [56]. Two significant risk factors for PTSD symptoms were identified in this study, namely female sex and high level of harm. The fact that females react more strongly is reported in other studies [46, 47]. Patel et al. [57] reported that work overload was the main contributor to ME. Residency was reported in literature as another risk factor. In fact, Bari et al. [58] reported that residents are a vulnerable population because residency is a learning period, and Abd Elwahab et al. [26] made it clear that junior physicians and residents are more prone to make MEs.

### Coping strategies

Few studies have investigated physicians’ needs and experiences in coping with the experience of error [18, 59]. Coping strategies used by “second victims” have a key role in how physicians involved in MEs will behave with their colleagues and subsequent patients. There are several different strategies for coping with the emotional impact after experiencing a ME. In this study, physicians used the following three coping strategies: problem-focused strategy, emotion-focused strategy, and seeking social support. These strategies are important for “second victims” to individually achieve an effective coping strategy through dealing with the ME, analyzing it, and learning from it, either alone or with colleagues. This finding was quasi in line with literature reporting that the two major used forms of coping are problem-focused coping and emotion-focused strategies [15, 59, 60]. In problem-focused coping, physicians try to cope with the problem that causes distress and try to solve it [15]. It aims to face up the mistake and address the problem directly. This finding was in line with the study of Harrison et al. [48] who reported that the most frequently and best coping method used was problem-focused strategy. In the emotion-focused strategy, physicians cope by managing the emotional distress caused by errors [60]. In seeking social support, individuals talk with family and friends in order to find emotional comfort. It is the less frequent coping strategy in this study, which is in line with a study reporting that talking about MEs to family and friends is less common [61]. The dynamic relationship between the impact and the coping strategy after an error is challenging to capture. In fact, the behavioral response to making a mistake may lead to the use of a particular coping strategy that, in return, may elicit a further behavioral response [62].

### Changes in practice

Experiencing a ME can cause considerable changes in medical practice [59]. These changes can be defensive or constructive [47]. Mizrahi et al. [63] described in a study conducted with internists in training, three defensive mechanisms to manage medical mistakes: denial process, discounting, and distancing. The findings in this study reveal that physicians considered that MEs more frequently lead to constructive changes. Communication and interaction with peers and superiors are perceived as the most helpful resources by 64.9 and 71.6% of participants, respectively. A minority of respondents (5%) reported defensive changes.

This study provided a quantitative analysis of the second victim phenomenon among Tunisian physicians. It shows for the first time the current situation in a North-African country. It clearly cut with the rather qualitative studies that previously addressed this topic. It also highlights the need for developing comprehensive organizational support strategies to help physicians cope with MEs. In the present study, despite the high impact of MEs, constructive changes concerned most respondents. One possible explanation may be that participants may have avoided reporting behavior seen as inappropriate and reported those that are generally considered socially desirable. In fact, MEs are still a taboo subject in developing countries, despite the growing body of literature on second victim phenomenon. This topic should be considered as an acute issue to be addressed by healthcare leaders. Raising awareness of second victim phenomenon and putting in place supportive programs is detrimental to improve clinician recovery and establish patient safety culture in the aftermath of a ME.

### Study limitations

This study has four limitations. First, it is possible that recall bias might have affected how physicians reported their past experiences. Further studies are needed to strengthen the present findings by continuously investigating ME and their impact during the clinical practice period. Second, over or under reporting cannot be entirely ruled out as a result of using of self-report questionnaires. It is true that in-depth interviews are more suitable for learning about MEs and their emotional impact; however, they could not be used because of the anonymous nature of the study. Third, this study included only physicians. It may be comprehensive to include other hospital employees (***eg***; nurses, midwives, pharmacists...) because they could be all concerned by MEs, and may be affected by stressful patient-related events. Finally, a ME is an annoying irony. This resulted in some people refusing to answer the questionnaire. In fact, the proportion of refusals could correspond to the most affected physicians.

## Conclusion

The “second victim” phenomenon is a potentially dangerous consequence of an unintentional error by healthcare professionals. The present study involving Tunisian University Hospitals’ physicians demonstrated the lack of recognition of this issue. The impact of medical errors was relatively high. This could lead to additional MEs and to further patient harm. Coping was balanced within respective three strategies as reported worldwide.

Future works are warranted to address the limitations by expanding the results of the present study as well as working on how hospitals should limit the negative effects of “second victim” experiences. This can be achieved through reducing punitive responses to MEs and encouraging supportive responses for physicians to cope with their involvement in MEs.

## Supplementary Information

**Additional file 1: Table S1.** Correlations between subscales of IES-R. **Table S2.** Correlations between subscales of WCC-R. **Table S3.** Convergent validity for IES-R. **Table S4.** Convergent validity for WCC-R.

## Data Availability

The datasets generated and/or analyzed during the current study are not publicly available due to limitations of ethical approval involving the participants’ data and anonymity but are available from the corresponding author on reasonable request.

## Abbreviations

IES-R: Impact of event scale-revised
MEs: Medical errors
PTSD: Post-traumatic stress disorder
SD: Standard deviation
WCC-R: Ways of coping checklist revised
