# Supplementary material for: North-African doctors as second victims of medical errors: a cross sectional survey
Source: BMC Psychiatry. 2022 Jun 20;22:411. doi: 10.1186/s12888-022-04049-0 (PMC9208235; doi:10.1186/s12888-022-04049-0)
Supplement: Supplementary file 1 — Additional file 1: Table S1. Correlations between subscales of IES-R. Table S2. Correlations between subscales of WCC-R. Table S3. Convergent validity for IES-R. Table S4. Convergent validity for WCC-R. [file 12888_2022_4049_MOESM1_ESM.docx]

**Content validity (redundancy)**

Correlations between subscales varied respectively between 0.722 and 0.771 for IES-R and between 0.416 and 0.516 for WCC-R (p=0.001). (S1 Table1, S2 Table2).

**S1 Table1:** Correlations between subscales of IES-R.

|  | **Intrusion** | **Avoidance** | **Hyperarousal** |
| --- | --- | --- | --- |
| **Intrusion** | **1** |  |  |
| **Avoidance** | 0.771 | **1** |  |
| **Hyperarousal** | 0.756 | 0.722 | **1** |

**S2 Table2:** Correlations between subscales of WCC-R.

|  | **Coping focused on the problem** | **Coping focused on emotions** | **Seeking social support** |
| --- | --- | --- | --- |
| **Coping focused on the problem** | **1** |  |  |
| **Coping focused on emotions** | 0.485 | **1** |  |
| **Seeking social support** | 0.516 | 0.416 | **1** |

**Convergent validity**

**S3 Table3:** Convergent validity for IES-R.

|  | Item 1 | Item 2 | Item 3 | Item 6 | Item 9 | Item 14 | Item 16 | Item 20 |
| --- | --- | --- | --- | --- | --- | --- | --- | --- |
| **Intrusion** | 0.653 | 0.649 | 0.7 | 0.638 | 0.675 | 0.699 | 0.742 | 0.484 |

|  | Item 5 | Item 7 | Item 8 | Item 11 | Item 12 | Item 13 | Item 17 | Item 22 |
| --- | --- | --- | --- | --- | --- | --- | --- | --- |
| **Avoidance** | 0.519 | 0.471 | 0.609 | 0.660 | 0.637 | 0.546 | 0.643 | 0.566 |

|  | Item 4 | Item 10 | Item 15 | Item 18 | Item 19 | Item 21 |
| --- | --- | --- | --- | --- | --- | --- |
| **Hyperarousal** | 0.508 | 0.633 | 0.667 | 0.388 | 0.534 | 0.517 |

**S4 Table4:** Convergent validity for WCC-R.

|  | Item 1 | Item 4 | Item 7 | Item 10 | Item 13 | Item 16 | Item 19 | Item 22 | Item 25 | Item 27 |
| --- | --- | --- | --- | --- | --- | --- | --- | --- | --- | --- |
| **Coping focused on the problem** | 0.391 | 0.351 | 0.444 | 0.260 | 0.646 | 0.411 | 0.396 | 0.376 | 0.504 | 0.554 |

|  | Item 2 | Item 5 | Item 8 | Item 11 | Item 14 | Item 17 | Item 20 | Item 23 | Item 26 |
| --- | --- | --- | --- | --- | --- | --- | --- | --- | --- |
| **Coping focused on emotions** | 0.520 | 0.264 | 0.510 | 0.386 | 0.525 | 0.227 | 0.421 | 0.505 | 0.380 |

|  | Item 3 | Item 6 | Item 9 | Item 12 | Item 15 | Item 18 | Item 21 | Item 24 |
| --- | --- | --- | --- | --- | --- | --- | --- | --- |
| **Seeking social support** | 0.389 | 0.483 | 0.525 | 0.554 | **-0.011**  **p=0.828** | 0.605 | 0.266 | 0.463 |

All WCC-R items seem convergent (p˂0.001), except for item 15.
